# Systematic review of clinical trials on dietary interventions to prevent excessive weight gain during pregnancy among normal weight, overweight and obese women

**DOI:** 10.1186/1471-2393-11-81

**Published:** 2011-10-26

**Authors:** Ida Tanentsapf, Berit L Heitmann, Amanda RA Adegboye

**Affiliations:** 1 Faculty of Health Sciences, Copenhagen University, Panum Instituttet, Blegdamsvej 3B, 2200, Copenhagen, Denmark; 2 Research Unit for Dietary Studies, Institute of Preventive Medicine, Copenhagen University Hospital, Oster Sogade 13, 1357, Copenhagen, Denmark

## Abstract

**Background:**

Excessive weight gain during pregnancy and subsequent postpartum weight retention may contribute to the epidemic of obesity among women of childbearing age. Preventing excessive gestational weight gain (GWG) to optimize maternal, fetal and infant wellbeing is therefore of great importance. A number of dietary interventions in this area has been conducted with inconsistent results, which has made it difficult to identify effective strategies to prevent excessive weight gain during pregnancy among normal weight, overweight and obese women. The primary objective of this review was to evaluate the effect of dietary interventions for reducing GWG. The secondary objective was to examine the impact of these interventions on different child and maternal health outcomes.

**Method:**

The PUBMED, the Cochrane Central Register of Controlled Trials (CENTRAL) and the LILACS databases were searched for relevant articles. All published randomized controlled trials (RCT) and quasi-randomized controlled trials (QCT), with concurrent controls, on dietary interventions during pregnancy were considered. Results were presented using relative risk (RR) for categorical data and weighted mean difference (WMD) for continuous data. Data were primarily analyzed with a fixed-effect model and a random-effects model was used in the presence of heterogeneity. No date and language restrictions were applied.

**Results:**

In total, 13 studies were included in this review and 10 trials contributed data on total GWG. Dietary intervention significantly reduced total GWG (n = 1434; WMD = -1.92 kg; 95% CI = -3.65/-0.19; p = 0.03), weight retention at six months postpartum (n = 443; WMD = -1.90 kg; 95% CI = -2.69/-1.12; p < 0.0001) and incidence of cesarean section (n = 609; RR = 0.75; 95% CI = 0.60/0.94; p = 0.013). However, dietary intervention had no significant effect on weight retention at six weeks postpartum, birth weight, preeclampsia, gestational diabetes and preterm birth.

**Conclusion:**

Dietary advice during pregnancy appears effective in decreasing total GWG and long-term postpartum weight retention, but so far there is limited evidence for further benefits on infant and maternal health.

## Background

One of the most common factors resulting in a shorter life expectancy and several diseases is obesity [1]. Strategies aimed at preventing weight gain and obesity have proven to be easier and less costly than those aimed at treating already obese people [1].

Targeting pregnant women has been the focus of dietary and lifestyle interventions [2], as one third of pregnant women tend to gain excessive weight during their pregnancy [3]. Several studies have shown that excessive weight gain during pregnancy is a strong predictor of postpartum weight retention [4] and this may contribute to obesity in women of childbearing age [5]. According to the Institute of Preventive Medicine (IOM) based on pre-pregnancy body mass index (BMI), normal weight women (BMI: 18.5-24.9) are recommended to gain between 11.4 and 15.9 kg during pregnancy, overweight women (BMI: 25.0-29.9) between 6.8 and 11.4 kg and obese women (BMI: ≥ 30) between 5.0 and 9.0 kg [6]. There is evidence suggesting that weight gains within IOM recommendations are potentially associated with healthy fetal and maternal outcomes [7].

Maternal obesity is associated with several negative pregnancy outcomes, including, hypertensive conditions, preeclampsia, gestational diabetes, required induction of labor, cesarean section, having a stillbirth, perinatal death, macrosomia (birth weight > 4000 g) preterm birth (< 37 weeks of gestation), congenital anomaly, increased risk of childhood obesity and development of type 2 diabetes [8]. Not only is maternal obesity associated with complications during pregnancy, but also excessive gestational weight gain (GWG) among normal weight women is a risk factor for negative pregnancy outcomes [9]. Therefore, achieving a healthy weight gain during pregnancy is an important issue for all women.

It is well known that during pregnancy the nutritional requirement is enhanced and that women in general attend this demand by increasing their food intake. However, cultural beliefs, such as "eating for two" may contribute to a caloric intake above the ordinary demands of pregnancy [10,11]. Since weight gain partially reflects an imbalance between energy intake and energy expenditure, it seems plausible that during prenatal care visits women should be motivated to change their lifestyle towards healthy dietary habits.

Additionally, such behavioural changes attained during pregnancy may persist after childbirth and possibly throughout the woman's life [11,12]. Therefore, healthcare providers should take advantage of these prenatal care visits as a window of opportunity for implementing effective lifestyle interventions during pregnancy.

Previous reviews have attempted to summarize the available evidence of dietary interventions in regard to GWG [2,13-16]. However, the reviews have showed some constraints and inconsistent results, which has made it difficult to identify effective strategies to prevent excessive GWG among normal weight and obese women. Kramer et al [14] included only three randomized controlled trials (RCTs) on caloric restriction; Dodd et al. [2,13] included nine RCTs that focused on interventions only for overweight and obese women. Three RCTs included women with gestational diabetes. Streuling et al. [16] included nine studies in total. Of these, five studies were not RCTs. Skouteris et al [15] included ten studies in total, three were not RCTs. Meta-analysis was not performed to summarize the results

Since non-RCTs tend to render stronger effect sizes than RCTs [17] the purpose of this study was, therefore to perform a systematic review with meta-analysis using high quality evidence of the published dietary interventions for preventing excessive GWG.

## Method

### Type of studies

RCTs and QCTs with a concurrent control group assessing the dietary effect on GWG were eligible for inclusion in this review.

### Type of participants

Healthy normal weight or overweight and obese women with a singleton pregnancy were eligible. Women under the age of 18 years were excluded in order to avoid the contribution of natural linear growth to GWG, as were women taking any type of medication that might interfere with their body weight (e.g. steroids, diuretics, thyroid hormones and amphetamines). Trials enrolling underweight or pregnant women with an increased risk of insufficient weight gain and or giving birth to low birth weight babies (e.g. women exposed to heavy manual labour) were also excluded

### Type of intervention

Any type of dietary intervention aiming at preventing excessive GWG or reducing pregnancy related complications (preeclampsia, gestational diabetes, macrosomia, cesarean section) was considered. There was no restriction in regards to the intensity, frequency and timing of the intervention as well as who preformed it (e.g. public health nurses, dietitians or physicians). This included low-fat, low-carbohydrate or low-energy diets as well as dietary education about healthy eating and nutritional advice on how to stay within the GWG guidelines. Interventions not specifically designed to prevent excessive GWG were excluded.

### Type of outcome measures

The primary outcomes were: percentage of women who gained weight above the IOM recommendations, or percentage of women with excess GWG (regardless of weight gain guidelines used), and total GWG or weekly GWG. The secondary outcomes were divided into maternal and infant outcomes. Maternal outcomes included: postpartum weight retention, preeclampsia, gestational diabetes and cesarean section. Infant outcomes included: birth weight; incidence of low birth weight (< 2500 g), high birth weight or macrosomia (> 4000 g), preterm birth and gestational age.

### Search strategy

The PUBMED, the Cochrane Central Register of Controlled Trials (CENTRAL) and the LILACS databases were used to search for relevant articles (last search conducted in March 2011) to be included in this systematic review (For full search strategy see Additional file 1: Appendix 1). No date and language restrictions were applied.

The PUBMED search retrieved 1358 articles. The title and abstract of each article were then reviewed to determine the eligibility for inclusion regarding type of study, participants, intervention and outcomes. In the presence of doubt about study eligibility for inclusion, the articles were included and the final decision was taken at the next stage. In the second stage, the full text of the article was obtained to clarify doubts about eligibility criteria. This resulted in 19 articles being retained and fully reviewed, but only 10 met the inclusion criteria [18-27]. Of the nine excluded articles, three were ongoing trials [28-30] and six intervention studies [31-36] were neither RCTs nor QCTs with concurrent controls.

The CENTRAL search retrieved 602 articles and after reviewing the titles and abstracts 11 articles were selected. Of these, 10 studies met the inclusion criteria, seven articles [18-20,23,25-27] were previously selected in the PUBMED search and only three articles [37-39] were finally retained. One article was excluded [40] due to use of drugs in combination with diet.

**Figure 1:**
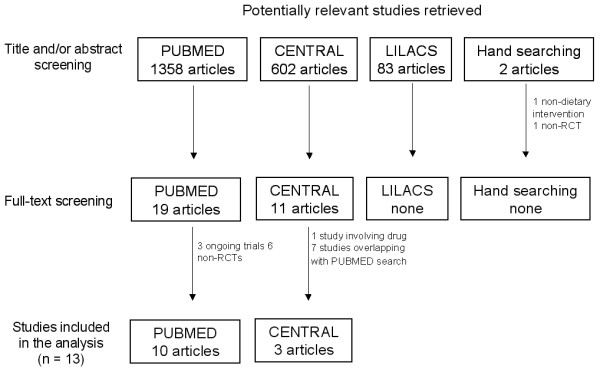
**Flow chart of the selection process for eligible studies**.

The LILACS search retrieved 83 articles and after reviewing the titles and abstracts no article was selected. Two studies [41,42] were identified after hand searching reference lists. However, none of them met the inclusion criteria (Characteristics of excluded Studies can be found in Additional file 1: Appendix 2). A total of 13 studies [18-27,37-39] were included in this systematic review (Figure 1).

### Data extraction

Using a standardized data extraction form, which was tested in a pilot study, we extracted all relevant information. When needed, further information or data were requested from trial authors. Two authors (IT & AA) extracted relevant information and evaluated the methodological quality of included trials independently. Differences in data extraction were resolved by consensus, referring back to the original article.

### Quality assessment

Studies were assessed according to five methodological features [43]. Studies were classified as randomized on the basis of the study report: (A) stated in the text; (B) unclear or not stated and (C) not used.

Regarding concealment of allocation studies were classified as: (A) adequate, assignment to groups was determined by central off-site randomization, sequentially numbered, sealed, opaque envelopes or other appropriate schemes and so could not be influenced by the investigators; (B) unclear or not stated; (C) inadequate, alternation, the use of case record numbers, dates of birth or day of the week, tossing a coin and any procedure that was entirely transparent before allocation and (D) not used.

Double blinding was impossible in these kinds of studies, as the participants knew which intervention they received. However, blinding of those assessing the results (single blinding) was considered. Studies were classified as: (A) adequate, the investigator who assessed the results did not know the allocated treatment; (B) unclear or not stated; (C) no blinding, the investigator knew the allocated treatment.

Regarding completeness of follow-up, studies were classified as: (A) adequate, less than 20% of withdrawal or loss to follow-up; (B) unclear or not stated; (C) inadequate, more than 20% of withdrawal or loss to follow-up. Based on the quality criteria, studies were broadly subdivided into the following three categories: (A) low risk of bias, all quality criteria met; (B) moderate risk of bias, one or more of the quality criteria only partly met; (C) high risk of bias, one or more criteria not met.

### Analysis

When data was available, sufficiently similar and of sufficient quality, statistical analyses using STATA (StataCrop 9.2, Texas) were performed. For continuous outcomes, results were expressed as weighted mean difference (WMD) between the post-intervention values, or the difference between baseline values and post-intervention values. For dichotomous outcomes, results for each study were expressed as relative risk (RR). Both dichotomous and continuous outcomes were presented with 95% confidence intervals (CI).

GWG can be estimated using different anthropometric indicators (e.g. total weight gain, weekly or monthly rate of gain) or as percentage of women who gained above the IOM recommendations. Therefore, the data were grouped and analysed according to the methods used to estimate GWG. When information was provided in the article, an intention-to-treat analysis was planned to be performed.

The individual studies were weighted by their inverse variances. Firstly, the data were analysed with a fixed-effect model. The I^2 ^statistic was applied to describe the proportion of total variation in study estimates that was due to heterogeneity. An I^2 ^of more than 50% was considered as notable heterogeneity. When high levels of heterogeneity were found, pre-specified subgroup and sensitivity analyses (e.g. type of intervention, type of participants and study quality, excluding trials most susceptible to bias) were performed. Metainf command was used to investigate the influence of a single study on the overall meta-analysis estimate. Whether pooling of results seemed appropriate, heterogeneity that was not explained by subgroup and sensitivity analyses, was modelled using a random-effects analysis, which assumes that the effect size varies across studies.

Funnel plot was used as a visual tool for investigating the presence of potential bias. If publication bias is not present, the plot is expect to be roughly symmetrical resembling an inverted funnel. To facilitate interpretation of the funnel plot, diagonal lines representing the 95% CI around the pooled estimate for each standard error (SE) on the vertical axis was added to the plot (WMD ± 1.96 SE). In the absence of heterogeneity or selection bias, 95% of the studies should lie within the funnel defined by these lines. Because these lines are not strict 95% CI, they are referred to as "pseudo 95% CI"[44].

## Results

### Description of studies

Of the 13 trials included in this review, 11 studies were carried out in Western countries [18-20,22-27,38,39], one in Egypt [37] and one in Taiwan [21]. Most studies recruited women who were in the first or second trimester of gestation [18,20-27,37,39] and two studies recruited women in the third trimester [19,38].

All trials involved a dietary intervention, but there were differences in the conduct. Seven were of a lifestyle changing nature, with diet and physical activity (PA) counseling [18,20,21,23-25,39]. Four trials had an additional treatment, such as: motivational phone calls, posted materials or brochures [20,21,24,25]. Two trials focused solely on reducing the amount of calories consumed [37,38], while five studies had additional guidelines for what the diet should consist of, regarding percentage of carbohydrates, protein and fat [18,19,22,26,27]. Six trials included education feedback relating to weight gain according to IOM guidelines [18,20,22-25]. Only two out of the 13 studies, did not have at least one face-to-face session with dietary counseling [19,37].

All studies, but four [19-22] included two comparison groups: diet or diet plus PA (intervention) vs. usual care (control). Guelinckx et al. [20] included three groups: diet (intervention 1), diet plus lifestyle (intervention 2) and usual care (control). Therefore, Guelinckx et al. was included twice in the analysis: comparison of diet vs. usual care and diet plus lifestyle vs. usual care. Ilmonen et al.[22] included comparisons among diet plus placebo (intervention 1) vs. diet plus probiotics (intervention 2) vs. usual care plus placebo (control). However, only comparison between diet plus placebo vs. usual care plus placebo was considered in this review. Campbell [19] included comparisons on diet (intervention 1), diuretics (intervention 2) and usual care (control). Data on diuretics was not considered. In Huang's trial [21] participants were assigned to either intervention 1 (from pregnancy to six months postpartum), intervention 2 (from birth to six months postpartum) or a control group. Since intervention 2 was restricted to postpartum period it was not considered in this review.

Phelan et al [24] and Polley et al [25] analyzed data of normal weight and overweight/obese (BMI > 25) women separately. However, combined results from both groups were presented in this review. Characteristics of the included studies are described in Table 1.

**Table 1:** Characteristics of included studies

Author/year	Country	Design	Population	Recruitment	Intervention type	Description	Quality	Risk of bias
Asbee, 2009 [18]	USA	RCT	n = 100 All BMI categories (< 40.5 kg/m^2^), age 18-49 years	wk 6-16 of gestation	1. diet + PA counseling 2. usual care	Individual session with a dietician only at 1^st ^visit. Diet should consist of 40% CH, 30% protein and 30% fat. GWG monitored at every visit. Moderate exercise 3-5 times/wk.	Randomization: A Allocation: A Blinding: B Losses: A	moderate

Badrawi, 1992 [37]	Egypt	RCT	n = 100 Obese multiparous women, age 25-35 years	early in pregnancy	1. caloric restriction 2. usual care	Usual care: Normal diet according to WHO energy recommendations (2300-3000 kcal/day). Intervention: balanced low-energy diet (1500-2000 kcal/day).	Randomization: A Allocation: B Blinding: B Losses: B	moderate

Campbell, 1975 [19]	Scotland	QCT	n = 102 Primiparous women with high GWG (> 570 g/wk) at 20-30 weeks gestation	wk 30 of gestation	1. caloric restriction 2. usual care 3. diuretics*	A low-energy diet (1200 kcal/day) with low CH. The second intervention group was excluded, due to use of drugs as part of the intervention.	Randomization: B Allocation: C Blinding: B Losses: B	high

Campbell 1982 [38]	Scotland	QCT	n = 182 Obese primiparous women	wk 29-30 of gestation	1. caloric restriction 2. usual care	A low-energy diet (1250 kcal/day), instructed by a dietitian at recruitment	Randomization: B Allocation: C Blinding: B Losses: B	high

Guelinckx, 2009 [20]	Belgium	RCT	n = 122 White, obese pregnant women, BMI > 29	< wk 15 of gestation	1. brochure 2. brochure + diet + PA counseling 3. usual care	Intervention 1: Given a purpose design brochure at 1^st ^prenatal consultation, with nutritional and PA advice to limit GWG according to IOM guidelines. Intervention 2: Brochure + active lifestyle education by a nutritionist in 3 1 hour group sessions. All participants: Nutritional habits evaluated every trimester with three 7-day food records.	Randomization: A Allocation: A Blinding: C Losses: C	high

Huang, 2009 [21]	Taiwan	RCT	n = 125 Pregnant women ≥ 18 years of age	< 16 wk of gestation	1. diet + PA counseling + brochure during pregnancy 2. diet + PA counseling + brochure given postpartum* 3. usual care	Usual care: Routine obstetric educational program, once each trimester. Intervention 1: 6 individual session with a dietician with individualized diet and PA plan + brochure, from recruitment to 6 months post partum.	Randomization: A Allocation: A Blinding: A Losses: C	high

Hui, 2006 [39]	Canada	RCT	n = 45 Pregnant women with no preexisting diabetes	< 26 wk of gestation	1. diet + PA counseling 2. usual care	Usual care: information package on diet and PA for a healthy pregnancy. Intervention: Group and home based exercises (3-5 times/wk for 30-45 min was recommended). They also received Computer assisted Food Choice Map, dietary interviews and counseling.	Randomization: A Allocation: B Blinding: B Losses: A	moderate

Ilmonen, 2010 [22]	Finland	RCT	n = 171	< 17 wk of gestation	1. diet + placebo 2. diet + probiotics* 3. usual care + placebo	Intervention groups: Dietary counseling (nutritionist) + probiotic or placebo capsules and food products for home use, each trimester and at 1, 6 and 12 months post partum. Diet should consist of 55-60% CH, 10-15% protein and 30% fat.	Randomization: A Allocation: A Blinding: A Losses: C	high

Kinnunen, 2007 [23]	Finland	QCT	n = 105 Normal weight primiparous women ≥ 18 years	< 8-9 wk of gestation	1. diet + PA counseling 2. usual care	Usual care: Primiparas are recommended 11-15 visits to a public health nurse and 3 to a physician during pregnancy. Intervention: Individual counseling on diet + PA and IOM guidelines for GWG, during 5 routine visits to a public health nurse from wk 8-9 to wk 37 of gestation. Option to attend supervised group exercise.	Randomization: D Allocation: B Blinding: B Losses: C	high

Phelan, 2011 [24]	USA	RCT	n = 358 Non-smoking pregnant women, BMI 19,8-40	wk 10-16 of gestation	1. diet + PA counseling 2. usual care	Intervention: Standard care + 1 visit to interventionist promoting self monitoring including; appropriate weight gain, PA (30 min/day) and diet (20 kcal/kg). Participants also received 3 phone calls from a dietitian + weekly mail.	Randomization: A Allocation: A Blinding: A Losses: A	low

Polley, 2002 [25]	USA	RCT	n = 110 Normal weight pregnant women, BMI 19,8-26 Overweight pregnant women, BMI > 26	< 20 wk of gestation	1. diet + PA counseling 2. usual care	Intervention: Regularly antenatal visits with access to research dietician and psychologist. Newsletters and phone calls between clinical visits, with education and feedback relating to weight gain, exercise and healthy eating.	Randomization: A Allocation: B Blinding: B Losses: A	Moderate

Thornton, 2009 [26]	USA	RCT	n = 232 Obese pregnant women, BMI ≥ 30	wk 12-28 of gestation	1. caloric restriction 2. usual care	Intervention: Placed on an 18-24 kcal/kg diet consisting of 40% CH, 30% protein, and 30% fat after a visit to a dietitian. The women were asked to record in a diary all of the foods and beverages consumed during each day.	Randomization: A Allocation: A Blinding: B Losses: A	moderate

Wolf, 2008 [27]	Denmark	RCT	n = 50 Caucasian obese pregnant women, BMI ≥ 30	wk 15-18 of gestation	1. caloric restriction 2. usual care	Intervention: Restriction of GWG to 6-7 kg by 10 1-hour dietary consultations with a trained dietitian, at each antenatal visit. Individual recommendation on daily energy intake, coming from 50-55% CH, 15-20% protein and max 30% fat, according to the official Danish dietary recommendations. 7 day weighed food records were used and individualized suggestions of improvement, were given to those with an identified unhealthy eating pattern.	Randomization: A Allocation: B Blinding: A Losses: C	high

BMI - body mass index, CH - carbohydrates, GWG - gestational weight gain, min - minutes, PA - physical activity, QCT - quasi-randomized controlled trial, RCT - randomized controlled trial, wk - week

* Comparison group not considered in this review

### Methodological quality

Randomization of participants into different groups was stated in 10 out of the 13 studies [18,20-22,24-27,37,39]. In six of the RCTs, the method of randomization was adequate [18,20-22,24,26], but in the remaining four the method used was not reported [25,27,37,39]. One study was an affirmed non-RCT [23], while in the remaining two this was unclear [19,38]. Allocation concealment was adequate in six of the trials [18,21,22,24,26]. In five trials the allocation process was unreported [23,25,27,37,39] and in the remaining two it was inadequate [19,38]. The completeness of follow-up was adequate in five trials [18,24-26,39], unreported in three [19,37,38] and inadequate in five [20-23,27]. Outcome data, collected by investigators blinded to group allocation, was applied in four trials [21,22,24,27].

### Primary outcomes

Table 2 shows the pooled estimate effect of dietary intervention on all outcomes. Ten trials [18,20-27,39] contributed data for the analysis of total GWG. Although Badrawi et al [37] stated in the abstract that there was a difference in weight gain between the diet versus the control group, no numeric results were presented.

**Table 2:** Pooled estimate effect of dietary intervention during pregnancy on different outcomes

Outcomes	Studies	Comparison groups	Participants	Statistical method	Effect size (95% CI)	I^2^
Total GWG						
all data	10	11	1434	WMD (Random)	-1.92 kg (-3.65/- 0.19)	89%
excluding Thornton and Wolff	8	9	1152	WMD (Fixed)	-1.01 kg (-1.58/-0.45)	43%

Weekly GWG	2	2	253	WMD (Random)	-0.26 kg/wk (-0.42/-0.09)	82%

GW above IOM guidelines	4	4	629	RR (Fixed)	0.90 (0.77/1.05)	0%

Weight retention						
6 wks postpartum	2	2	306	WMD (Fixed)	0.58 (0.13/1.03)	12%
6 mths postpatum	3	3	443	WMD (Random)	-1.90 (-2.69/-1.12)	63%

Preeclampsia	6	6	1025	WMD (Fixed)	0.78 (0.58/1.06)	0%

Gestational diabetes	6	6	886	WMD (Fixed)	0.74 (0.52/1.06)	31%

Cesarean section						
all data	6	6	841	RR (Random)	0.82 (0.60/1.09)	61%
excluding Thornton	5	5	609	RR (Fixed)	0.75 (0.60/0.94)	0%

Mean birth weight						
all data	7	8	1048	WMD (Random)	-34.8 g (-162.6/93.0)	77%
excluding Badrawi	6	7	949	WMD (Fixed)	34.5 g (-27.4/93.5)	0%

Low birth weight	2	2	531	RR (Fixed)	1.30 (0.8/2.10)	0%

Macrosomia	6	6	1023	RR (Fixed)	0.94 (0.62/1.35)	33%

Mean gestational age	7	8	1167	WMD (Fixed)	0.22 (0.01/0.42)	0%

Preterm birth	4	4	873	RR (Fixed)	0.83 (0.51/1.34)	0%

CI - confidence interval, GWG - gestational weight gain, IOM - Institute of Preventive Medicine, wks - weeks, mths - months WMD - weighted mean difference, RR - relative risk

**Figure 2:**
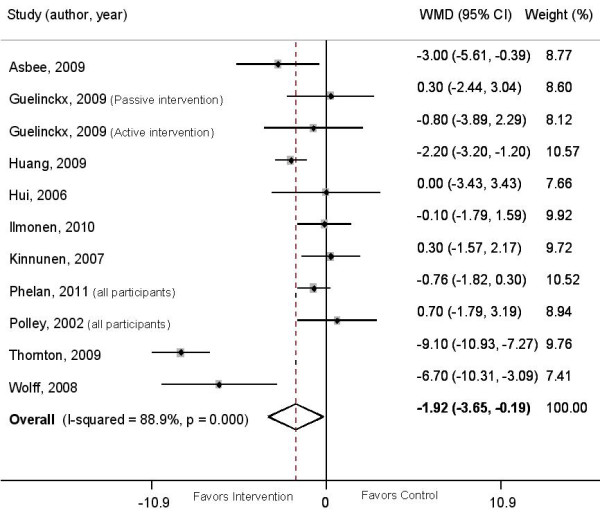
**Weighted mean difference in total gestational weight gain between intervention and control groups**. The overall effect size was estimated by weight mean difference using inverse variance method. Weights are from random effects analysis. The black dot represents the point estimate of each study, square size represents the weight of each study in the meta-analysis and the horizontal lines represent the respective 95%CI. The vertical solid line represents WMD of zero or line of no effect. The diamond represent the overall pooled estimate effect of the dietary intervention.

A significant heterogeneity was found in the meta-analysis of total GWG (I^2 ^= 89%; p < 0.0001). Subgroup analyses according to the type of intervention (caloric restriction vs. nutritional counselling; and individual vs. group counselling), type of participants (all BMI categories vs. overweight and obese women) and study quality did not explain the heterogeneity. Therefore, a random-effects model was used. Figure 2 shows a lower total GWG in the intervention group compared to the control group (WMD = -1.92 kg; 95% CI = -3.65/-0.19; p = 0.03). The study by Thornton et al [26] and Wolff et al [27] showed the strongest effect estimate of all included studies. Simultaneous exclusion of both trials [26,27] reduced the heterogeneity to I^2 ^value of 43% (p = 0.08) and the overall effect size to -1.0 kg (95% CI = -1.58/-0.45).

The data on weekly GWG, provided by two trials [19,38] showed significant heterogeneity (I^2 ^= 84.8%; p = 0.02). Influential, sensitivity and subgroup analyses were not possible due to the limited number of studies included. Therefore, a random-effects model was used. Both trials [19,38] reported that caloric restriction in obese women or women with a high weight gain was associated with a significant reduction in weekly GWG (WMD: -0.26 kg/wk; 95% CI -0.42/-0.09; p = 0.003).

Only four trials [18,23-25] contributed data for the comparison of percentage of women who gained weight above the IOM recommendations between groups. The pooled estimate showed a trend towards reduction in risk of gaining excessive weight (RR = 0.90; 95% CI = 0.77/1.05). However, the result was non-significant (p = 0.18). No heterogeneity was found in these data. One additional trial [39] reported data on excessive GWG according to the Canadian GWG guidelines [45]. Inclusion of Hui's [39] trial in the pooled estimate did not alter the results (RR = 0.89; 95% CI = 0.77/1.04, data not shown).

### Secondary outcomes

Huang et al [21] and Wolff et al [27] (unpublished data) reported weight retention at six months postpartum. Phelan et al [24], Polley et al [25] and Thornton et al [26] reported weight retention at six weeks postpartum. In all studies, postpartum weight was either measured by a research assistant or by a gynecologist during the routine postpartum visit. Dietary intervention had no significant effect on weight retention at six week postpartum. However, weight retention was significantly lower in the intervention group compared to control group at six months postpartum. Data showed significant heterogeneity.

Six trials reporting occurrence of preeclampsia [19,24-27,38] and gestational diabetes [24-27,37,39] were identified. No significant decrease in the incidence of preeclampsia and gestational diabetes was found. Six trials [18,24-27,37] had available data on cesarean section and the data showed significant heterogeneity (I^2 ^= 61%; p = 0.02). The heterogeneity was explained by the influence analysis omitting the study by Thornton et al.[26], which showed an increased risk of cesarean section among the intervention group. The pooled estimate including the remaining studies [18,24,25,27,37] showed a significant decrease in the incidence of cesarean section in the intervention group compared to the control group.

Seven trials [19,20,22,24,27,37,39] contributed data on mean birth weight. The data showed significant heterogeneity (I^2 ^= 77%; p < 0.0001). The heterogeneity was explained by the influence analysis omitting the study by Badrawi et al [37], which involved a balanced low-energy diet (1500-2000 Kcal/day). In the trial by Badrawi et al [37], infants weighed significantly less in the intervention group compared to the control group (3500 g vs. 3950 g, *p *< 0,001). However, the pooled estimate including the remaining studies [19,20,22,24,27,39] showed that dietary intervention had no effect on mean birth weight. Subgroup analysis including only interventions based on caloric restriction [24,27,38,39] did not significantly reduce the mean birth weight (WMD = -127.6; 95% CI = -353.6/98.5; data not shown). Only two trials [24,38] contributed data on low birth weight. No significant increase in the incidence of low birth weight was found in the intervention group compared to control group. Six trials [23-26,38,39] contributed data on macrosomia. Dietary intervention did not significantly decrease the incidence of macrosomia.

Eight trials reported results on mean gestational age [20,22,24-27,38,39]. Polley et. al [25] provided data on mean gestational age for control and intervention group, but SDs were not available and therefore this trial was not included in the statistical analysis. Four trials contributed data on preterm birth [24,26,38,39]. Dietary intervention slightly increased the duration of gestation in 0.22 weeks (95% CI = 0.01/0.42) compared to the control group. However, the intervention did not significantly reduce the risk of preterm birth (RR = 0.83; 95%CI = 0.51/1.34). Analysis restricted to caloric restriction trials reporting mean gestational age [24,26,27,38] did not result in a clinically important shortening of gestation (WMD = 0.19 weeks; 95%CI -0.07/0.45, data not shown).

### Publication bias

**Figure 3:**
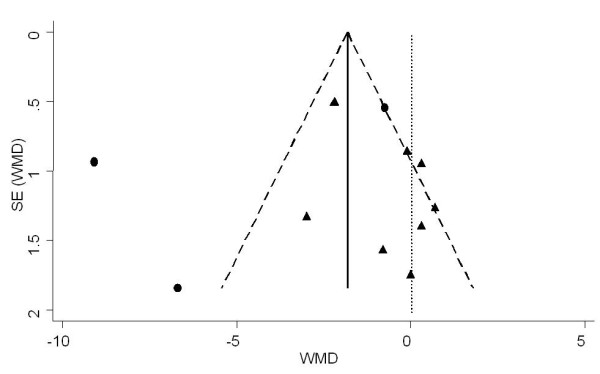
**Funnel plot of the SE by weighted mean difference (WMD) using random effect model for assessment of publication bias**. The vertical solid line represents the pooled estimate (WMD) and the diagonal dashed lines represent the pseudo 95%CI around the pooled estimate. The vertical dotted line represents the WMD of zero or line of no effect. Each circle represents a study applying caloric restriction and each triangle is a study not applying caloric restriction.

**Figure 4:**
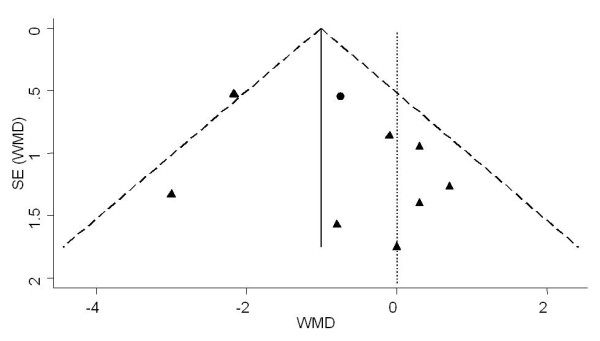
**Funnel plot of the SE by weighted mean difference (WMD) using fixed effect model for assessment of publication bias**. The vertical solid line represents the pooled estimate (WMD) and the diagonal dashed lines represent the pseudo 95%CI around the pooled estimate. The vertical dotted line represents the WMD of zero or line of no effect. Each circle represents a study applying caloric restriction and each triangle is a study not applying caloric restriction.

The first plot (Figure 3) was performed using all 10 studies reporting data on GWG [18,20-27,39]. The analysis revealed no apparent publication bias. Studies with significant results did not appear to be superior to studies with null results with respect to quality of design. There was no gap in the right bottom side of the graph (around no difference) indicating that smaller studies showing no statistically significant effects were included in the review. No trend towards overestimation of treatment effects in smaller studies of lower methodological quality was observed. However, clinical heterogeneity was evident, with studies applying caloric restriction [26,27] showing higher effect sizes (Figure 3). After exclusion of trials by Wolff et al [27] and Thornton et al [26] the plot was relatively symmetric (Figure 4).

## Discussion

This systematic review indicates that dietary intervention during pregnancy appears effective to reduce total and weekly GWG and incidence of cesarean section. However, there was no significant evidence for effects on preventing excessive GWG (GWG above IOM guidelines). In addition, dietary intervention during pregnancy seems to have a significant effect on reducing long-term postpartum weight retention, but no effect on weight retention at 6 weeks after birth. However, early postpartum weight might be less important for the prediction of maternal obesity as weight at 6 weeks postpartum may be affected by edema, morphological stage of reproductive organs and lactation compared to 6-12 months [46].

Total GWG was significantly reduced by almost 2 kg in all types of interventions, indicating a clinically relevant reduction. Clinical heterogeneity was prominent across trails, particularly when looking at the characteristics of the participants, but also the type, the duration and the intensity of the interventions. As for the statistical heterogeneity that was identified, a random effects model analysis was applied when comparing studies. Thornton et al [26] and Wolff et al [27] showed the strongest effect estimate. Both trials included obese women recruited around their second trimester. The interventions applied were similar in terms of food composition, giving the women individual recommendations on daily energy intake and using food records to detect unhealthy eating. The intensity of the interventions varied markedly; one [27] being intense with 10 sessions of one-hour dietary consultations with a trained dietitian, at each antenatal visit and the other [26] being more of a monitoring nature, starting after the first visit to the dietician. Using food record might be time consuming, but appears important in reducing GWG as women become more aware of the foods they eat. However, total GWG was significantly reduced by 1 kg even after exclusion of the trials by Thornton et al [26] and Wolff et al [27] from the meta-analysis.

Weekly GWG was significantly reduced by 0.26 kg/wk in studies using caloric restriction, only. Both of the Campbell et al. trials [19,38] succeeded in reducing weekly maternal weight gain, although the magnitude of the reduction was markedly larger in the 1975 trial [19] where the caloric restriction was applied between 30 and 38 weeks' gestation. Women in the intervention group tended to have smaller babies than what would be expected in individual who had a high weight gain between 20 and 30 weeks' gestation. It therefore puts a question to whether dieting, late in pregnancy, might affect the weight gain of the fetus.

It is still unclear whether dietary interventions, particularly low-energy diet may increase the incidence of low birth weight since the overall effect of two caloric restriction trials was toward increased risk (RR = 1.30), but the confidence intervals were wide probably due to the limited number of studies included [24,38]. Subgroup analysis including only 4 trials based on caloric restriction [24,27,38,39] did not significantly reduce the mean birth weight, but likewise the overall effect was toward reduced mean birth weight (WMD = -127.6).

### Intervention targeting the high-risk group

Polley et al [25] and Phelan et al [24] delivered the same intervention to normal weight and overweight/obese women. However, both trials, based on behavioral lifestyle intervention, managed to reduce the frequency of excessive weight gain according to the IOM recommendation only in normal weight women, while the intervention had no significant effect on overweight/obese women and the trend being in the opposite direction. On the other hand, the trials by Wolff et al [27] and Thornton et al [26], based on caloric restriction, targeting overweight/obese women were effective in reducing total GWG in the intervention group. This might indicate that a low intensity behavioral intervention aiming to decrease high-fat foods and increase PA may not be sufficient to prevent excessive GWG in high-risk women (overweight/obese). More intensive interventions involving frequent contacts (e.g., weekly nutritional counseling) and emphasis on caloric restriction (18-25 kcal/kg) seems to be more appropriate for preventing excessive GWG among the overweight/obese women.

### Comparison with previous studies

A previous review [16], including four RCTs and five non-RCTs with either historical or concurrent controls, similarly indicated that the GWG was significantly lower in the intervention group compared to control. However, when the analysis was confined to the RCTs the results were no longer significant and only a trend to lower GWG in the intervention group was observed. This finding confirms the postulated overestimation of treatment effects by non-randomized trials. Nevertheless, the overall effect of our study was higher than that reported by Streuling et al [16] (reduction of 1.2 kg). This might be explained by the inclusion of new trials, particularly the ones by, Huang et al (-2.2 kg) [21], Thornton et al (-9.1 kg) [26] and Wolff et al (-6.7 kg) [27] in the present meta-analysis, which showed significant reduction in GWG. Another review by Dodd et al [13] included nine RCT, four not being specifically designed to prevent excessive GWG, but the review did not present any statistical significant data on the outcome measures.

### Strengths and limitations

To date, this study is the largest systematic review including 1434 normal weight and overweight/obese women, from dietary interventional studies with available information on total GWG. This systematic review was restricted to RCTs and QCTs in order to assure comparability between interventions and control groups, and to reduce risk of bias.

It was not possible to quantify the intensity of different interventions due to lack of details provided in the articles. Furthermore, problems with confounding were detected when looking at the methodological quality. Only one out of the 13 studies [24] had a final classification of low risk of bias, five [18,25,26,37,39] had a moderate risk and seven [19-23,27,38] had a high risk of bias.

As discussed in previous reviews; comparing GWG can be problematic as there is no common standard for calculations [15,16]. Among the reviewed studies, 10 studies calculated GWG based on self-reported pre-pregnancy weight [18,20-27,39] and three studies did not report the means of data collection when calculating GWG [19,37,38]. The final weight was taken at the day of delivery in four studies [18,20,21,26], and at the last clinic visit prior to delivery in six studies [22-25,27,39].

Another limitation was the lack of statistical power to capture small intervention effects on some clinical outcomes. The overall estimates tended to show that dietary intervention may reduce the incidence of preeclampsia, gestational diabetes and macrosomia, but the results did not reach statistical significance. In addition, there was lack of refined information on infant outcomes. None of the trials had available information on intrauterine growth restriction or small for gestational age. It would be relevant to assess the effect of dietary interventions on subcategories of preterm birth, such as moderately preterm birth (< 37 weeks) vs. very preterm birth (< 32 weeks) or spontaneous preterm birth vs. medically induced birth. However, only 4 trials contributed data on preterm birth [24,26,38,39] and none of them presented data on subcategories of preterm birth.

### Implications for practice and research

Dietary intervention seems to have no adverse effect on infant birth weight and gestational duration, but we could not find strong evidence that dietary intervention significantly reduced the incidence of preeclampsia, gestational diabetes and macrosomia. Further implications for fetal, infant, or maternal health cannot be judged from the available trials. Therefore, further research, with larger sample size, is required to confirm the results. Due to low methodological quality of included studies, future trials should ensure strict and concealed randomization, intention-to-treat analysis, and adequate blinding of outcome assessment. Since adherence to weight-control programs requires considerable effort, more information is necessary on women's satisfaction and compliance with such interventions. These outcomes should be evaluated in a systematic fashion.

It is suggested that dietary interventions targeting overweight/obese women should be more intensive than interventions targeting normal weight women. However, it was not possible to systematically quantify the intensity of interventions across trials and uncertainty on optimal intensity, limits the ability to generate reliable recommendations for clinical practice. It seems that nutritional counseling based on face-to-face visits and recommendation for patient-focused caloric intake, are more likely to be successful. Nevertheless, the ability of health care systems to deliver time-intensive interventions at population level remains unknown.

Informing and educating women on appropriate weight gain before and in the beginning of pregnancy, might contribute to a better compliance. Studies should focus more on the psychological aspect to why women are overweight to begin with. Women who are heavier before pregnancy are more susceptible to increase their weight gain during pregnancy and adhere less to IOM guidelines [47], while women who exercise, watch their dietary intake and weight before pregnancy, might be more likely to focus on staying within the IOM guidelines. Other pregnancy related weight gains, which need to be addressed, might depend on the lifestyle changes that accompany motherhood, leaving the women more vulnerable to eating disorders [48]. Not to forget, women who are pregnant are probably more likely to make healthier lifestyle changes, than during any other time in their life. Positive health outcomes should be lifted as well as negative outcomes, which are associated with not following guidelines in motivating women.

Previous observational studies have stated that family members might influence women on their exercise and dietary behavior during and after pregnancy and that the most normative influence was from their partners [49]. For this reason, future studies may consider taking into account the participation of family members, such as the husband or partner, as one of the characteristics of the intervention.

## Conclusion

In conclusion, dietary advice during pregnancy appears effective in decreasing total GWG and long-term postpartum weight retention. Unfortunately, the available data are insufficient to infer important risks or other potential benefits for the mother or infant. Studies investigating the cost-benefits for the health care system in implementing interventions for reducing total GWG between 1 kg - 2 kg on long-term outcomes are still needed.

## List of abbreviations

**BMI**: body mass index; **CH**: carbohydrates; **CI**: confidence interval; **GWG**: gestational weight gain; **min**: minutes; **mths: **months; **PA**: physical activity; **QCT**: quasi-randomized controlled trial; **RCT**: randomized controlled trial; **RR**: relative risk; **SE: **standard error; **wk**: week; **WMD**: weighted mean difference.

## Competing interests

The authors declare that they have no competing interests.

## Authors' contributions

IT and AA conceived the idea of the review and were in charge of study selection, quality assessment of studies and data extraction. AA was responsible for the data analysis. IT was in charge of drafting the study protocol and writing the final report. All authors read and approved the final manuscript.

## Pre-publication history

The pre-publication history for this paper can be accessed here:

http://www.biomedcentral.com/1471-2393/11/81/prepub

## Supplementary Material

Additional file 1 **Appendices 1 and 2**. Appendix 1 (description of electronic search strategy) and Appendix 2 (description of excluded studies). Click here for file
