# Supplementary material for: Systematic review of clinical trials on dietary interventions to prevent excessive weight gain during pregnancy among normal weight, overweight and obese women
Source: BMC Pregnancy Childbirth. 2011 Oct 26;11:81. doi: 10.1186/1471-2393-11-81 (PMC3215955; doi:10.1186/1471-2393-11-81)
Supplement: Additional file 1 — Appendices 1 and 2. Appendix 1 (description of electronic search strategy) and Appendix 2 (description of excluded studies). [file 1471-2393-11-81-S1.DOC]

**Additional file 1**

**Apendix 1: Search strategy**

**PUBMED search strategy**

#1 Type of study

randomized controlled trial[Publication Type] OR controlled clinical trial[Publication Type] OR clinical trial[Publication Type] OR Meta-analysis[Publication Type] OR Review [Publication Type] OR randomized controlled trials[MeSH Terms] OR random allocation[MeSH Terms] OR single blind method[MeSH Terms] OR clinical trials[MeSH Terms] OR Intervention Studies[MeSH Terms] OR comparative study[Publication Type] OR evaluation studies[Publication Type] OR follow-up studies[MeSH Terms] OR prospective studies[MeSH Terms] OR Clinical Trial*[Title/Abstract] OR Controlled Trial*[Title/Abstract] OR Intervention*[Title/Abstract] OR Randomised[Title/Abstract] OR Randomized[Title/Abstract] OR quasi-randomized[Title/Abstract] OR quasi-randomised [Title/Abstract] OR Pilot study [Title/Abstract]

#2 Type of participants

women[Title/Abstract] OR pregnancy[Title/Abstract] OR pregnant[Title/Abstract] OR gestation*[Title/Abstract] OR mother*[Title/Abstract] OR maternal[Title/Abstract] OR childbearing[Title/Abstract] OR pregnancy[MeSH Terms] OR Pregnant women[MeSH Terms]

#3 Type of intervention

diet[Title/Abstract] OR dieting[Title/Abstract] OR dietary[Title/Abstract] OR nutrition*[Title/Abstract] OR energy[Title/Abstract] OR calori*[Title/Abstract] OR lifestyle[Title/Abstract] OR life-style[Title/Abstract] OR life style[Title/Abstract] OR behaviour*[Title/Abstract] OR behaviour*[Title/Abstract] OR behavioral intervention [Title/Abstract]

#4 Outcomes

excessive weight [Title/Abstract] OR gestational weight[Title/Abstract] OR gestational weight gain [Title/Abstract] OR pregnancy weight [Title/Abstract] OR weight gain [Title/Abstract] OR weight-gain[Title/Abstract] OR weight retention [Title/Abstract] OR postpartum weight[Title/Abstract] OR post-partum weight [Title/Abstract] OR post partum weight[Title/Abstract] OR Institute of Medicine [Title/Abstract] OR IOM [Title/Abstract]

#5 Exclusions

fertility treatment*[Title] OR Neural tube defect*[Title/Abstract] OR Cancer*[Title] OR Carcinoma*[Title] OR Polycystic Ovary Syndrome[Title] OR Rat*[Title] OR Mouse[Title] OR Mice[Title] OR Cow*[Title] OR Sheep*[Title] OR Lamb*[Title] OR Men[Title] OR Male*[Title] OR Twin*[Title] OR Adolescent*[Title] OR Teen*[Title] OR Elder*[Title] OR postmenopause*[Title] OR post-menopause*[Title] OR menopause[Title]

#6 (#1 AND #2 AND #3 AND #4) NOT #5

**CENTRAL search strategy**

**pregnancy* OR pregnant OR gestation* OR gravid* OR mother* OR maternal OR childbearing** in **Title, Abstract or Keywords** and **diet* OR nutrition* OR energy OR calorie* OR lifestyle OR life-style OR life style OR behaviour* OR behaviour* OR behavioural intervention in Title, Abstract or Keywords**

AND

**excessive weight OR gestational weight OR gestational weight gain OR pregnancy weight OR weight gain OR weight-gain OR weight retention OR postpartum weight OR post-partum weight OR post partum weight in Title, Abstract or Keywords**

NOT

**Iron OR Supplement* OR AIDS OR HVI OR Adolescent* OR teen* Alcohol OR Tobacco OR Smoking OR Metformin* OR Massage OR Enteral OR Parenteral** **in Record Title**

**LILACS search strategy**

( ( ( ( ( rand ) OR "CLINICAL TRIAL" ) OR "COMPARATIVE STUDY" ) OR "CONTROLLED CLINICAL TRIAL" ) OR "META-ANALYSIS" ) OR "RANDOMIZED CONTROLLED TRIAL" [Publication type] AND women OR gravi$ OR gesta$ OR pregnan$ OR maternal$ OR mother$ OR female$ [Title words] and fat$ OR energ$ OR calori$ OR carbohydrate$ OR diet$ OR diet-therapy OR Nutri$ OR lifestyle OR life-style [Title words]

**Apendix 2:** Characteristics of excluded trials

| **Author, year** | **Reason for exclusion** |
| --- | --- |
| Althuizen, 2006 | Ongoing trial. No preliminary results on gestational weight gain provided |
| Breslow, 1963 | Use of drug (e.g. D-amphetamine sulphate and meprobamate) in combination with diet |
| Gray-Donald, 2000 | Inadequate study design (prospective intervention with historical control) |
| Knight, 2010 | Ongoing trial. No preliminary results on gestational weight gain provided |
| Lindholm, 2010 | Inadequate study design (intervention study without control group) |
| Mottola, 2009 | Inadequate study design (prospective intervention with matched control group). Controls were retrospectively selected from a large local perinatal database from women who delivered singleton births in same region during the same period as the intervention group (matched for pre-pregnancy BMI, maternal age, and parity) |
| Olson, 2003 | Inadequate study design (prospective intervention with historical control) |
| Rhodes, 2010 | Inadequate study design (no inclusion of a control group receiving standard pre-natal care). Overweight or obese pregnant women were randomly assigned to receive a low- glycemic load or a low-fat diet. |
| Shirazian, 2009 | Inadequate study design (prospective intervention with historical control matched for pre-pregnancy BMI, parity and socio-economic status) |
| Smith, 2010 | Ongoing trial. No preliminary results on gestational weight gain provided |
